# Pickling of chanterelle *Cantharellus cibarius* mushrooms highly reduce cadmium contamination

**DOI:** 10.1007/s11356-017-9819-2

**Published:** 2017-08-01

**Authors:** Małgorzata Drewnowska, Anetta Hanć, Danuta Barałkiewicz, Jerzy Falandysz

**Affiliations:** 1 Laboratory of Environmental Chemistry and Ecotoxicology, Gdańsk University, 63 Wita Stwosza Str, 80-308 Gdańsk, Poland; 2 Laboratory of Trace Element Analysis by Spectroscopy Method, Adam Mickiewicz University, Umultowska 89b, 61-614 Poznań, PL Poland

**Keywords:** Heavy metals, Mushroom, Food technology, Environmental pollution, Risk assessment

## Abstract

Mushrooms are considered as potential bio-remediation agents in soil polluted with heavy metals, while many species which efficiently accumulate them in flesh are edible. Question is if there is any possible culinary use of edible mushrooms with high heavy metal contents? This study aimed to investigate and discuss a fate of cadmium (Cd) in common household-treated fruitbodies of common chanterelle *Cantharellus cibarius*. The samples of *Cantharellus cibarius* Fr. were collected from five spatially distanced sites in Poland in 2011–2012. We examined from 267 to 358 fruiting bodies per collection, and in total 1565 fruiting bodies were used. Cadmium in fungal materials from all treatments and processes (mushrooms dried, deep frozen, blanched and pickled) was determined using validated methods by inductively coupled plasma mass spectrometry with dynamic reaction cell. Blanching of fresh chanterelles caused decrease of Cd by around 11 ± 7 to 36 ± 7%, while blanching of deep-frozen mushrooms by around 40 ± 6%. A rate of Cd decrease in chanterelles was similar when the fruiting bodies were blanched for 5 or 15 min and when used was potable or deionized water. Pickling of blanched chanterelles with a diluted vinegar marinade had a pronounced effect on further removal of Cd. Blanched chanterelles when pickled lost an extra 37–71% of Cd. Total leaching rate of Cd from fresh or deep-frozen fruitbodies of chanterelle when blanched and further pickled was between 77 ± 7 and 91 ± 4%. Blanching and pickling highly decreased content of Cd in *C*. *cibarius*.

Different species of mushrooms (basidiomycetes) from the wild and cultivated due to specificities in their physiology and/or an element abundance in a substrate may accumulate at elevated concentrations or hyper-accumulate (high values of bio-concentration factor) in fruitbodies the toxic elements, e.g. Ag, Cd, Hg, Pb or As (Borovička et al. 2007; Falandysz 2017; Falandysz and Rizal, 2016; Falandysz et al. 1994; Mleczek et al. 2015b). Also Cu and Zn, which are essential bio-elements, can be well bio-concentrated by mushrooms in fruitbodies (Kojta et al. 2016). For a mushroom enriched with a heavy metal, an option can be culinary use in aims to decrease contamination if fulfilled are the health safety conditions.

Accumulation or hyper-accumulation of a heavy metal by the mushroom may imply on possible usefulness of a species in mycoremediation technology of polluted soils. In context of mycoremediation, *Agaricus urinascens* (former name *Agaricus macrosporus*) and *Coprinus comatus* were suggested as possible candidates due to their capacity for bio-concentration of Cd, Cu, Hg and Pb (Cen et al. 2012; Falandysz 2016; García et al. 2005). An ability of a given species of mushroom to absorb by mycelium an element from a soil substrate and accumulate it in fruitbodies can be enhanced by an external application of a chelating agent (Cen et al. 2012). So far is unknown any example of a molecular modification aiming to increase efficiency of heavy metal absorption and sequestration in fruitbody by modification of a gene encoding the transporter or binding molecules. The metallothionein-like ligands were identified for Ag, Cd or Cu in some mushrooms by Münger and Lerch (1985) and Osobová et al. (2011). As a binding agent of Cd in *Agaricus urinascens* (former name *Agaricus macrosporus*), mycophosphatin was identified—a sulphur-free, non-metallothionein-like ligand (Schmitt and Meisch, 1985).

The question arise if there is any possible culinary use of edible mushrooms with high heavy metal content in flesh, e.g. species-specific accumulators, foraged in areas with geochemical anomaly, affected by anthropogenic emissions or harvested from the mycoremediation process? For example, the mushroom *Suillus variegatus* (common name variegated bolete) under a typical environmental condition can accumulate Fe in fruitbodies at up to 0.41% dry biomass (Falandysz et al. 2001). A scale of an enrichment that could be attractive for recovery of any noble or rare earth element but certainly not of Fe that is too common.

Cadmium apart from the Ag, Hg or Pb can be well accumulated and high in certain species of mushrooms that are grown in backgrounds (Árvay et al. 2014; Brzezicha–Cirocka et al. 2016; Falandysz et al. 2003, 2007a, b and 2017b; García et al. 2009; Kojta et al. 2016; Melgar et al. 1998 and 2016; Mędyk et al. 2017; Stefanović et al. 2016) or polluted areas, and also if grown in agroindustrial wastes (Falandysz 2017; Favero et al. 1990; Mleczek et al. 2015a). Cadmium is one of the priority inorganic environmental and food contaminants. Cooking has a potential to reduce the levels of Cd or other heavy metal in the mushrooms to a level low enough for safe consumption.

Deep freezing, thawing, slicing, drying, re-soaking or drying—grinding—macerating as well as acidification or the presence of chelating agents increase the leaching of Mn, Fe, Zn and Cu from blanched champignon *Agaricus bisporus*, while there is a deficit of the data for Cd (Biekman et al. 1996; Coskuner and Özdemir, 1997 and 2000; Drewnowska et al. 2017).

Blanching and pickling are traditional cooking methods for many mushrooms, and this study aimed to examine if blanching and pickling can reduce Cd in consumed mushrooms (chanterelles). An insight into a fate of Cd in processed and culinary-treated mushrooms can matter from a general food safety issue point of view regarding their nutritional and potential risk aspects and also for their potential use in bio-remediation or mycoremediation technology.

## Materials and methods

### Mushroom collection, preparation, household treatment and analysis

The samples of *Cantharellus cibarius* Fr. were collected from five spatially distanced sites (from 267 to 358 fruiting bodies per collection, and in total 1565 fruiting bodies were used) in Poland in 2011–2012. The sampling sites were the background areas at the locations: Jastrzębia Góra, Darżlubska Wilderness, Łapino, Tucholskie Pinewoods and Kościerzyna forest in the north-central region of Poland (Falandysz and Drewnowska, 2017). Traditionally, mushrooms were collected with bare hands and stored shortly in wooden baskets. Fruitbodies were rinsed with cold tap water and drained. Next, each fruitbody in collection from a particular site was sliced into four or three parts (vertical cuts using a plastic knife), which were pooled accordingly. In total, 36 composite subsamples (separated per site) were prepared from those mushrooms for experiments.

### Household treatment

Several experiments have been performed to examine an impact of processing and culinary treatment on fate of Cd in chanterelle (dried, frozen, blanched and pickled) mushrooms. A subsample of fresh and deep-frozen mushrooms from three locations were dried conventionally in an electrically heated dryer or freeze-dried, and further ground and subjected for Cd analysis.

Deionized water free of Cd, potable bottled water (Fonte spring from the location Nieszawa in Poland) free of a detectable Cd and a commercial spirit vinegar (acidity 10%) were used as media in the blanching and pickling experiments.

### Conventional drying

From each sample set, the subsamples of fresh individuals were selected. Mushrooms were placed into plastic trays of an electrically heated commercial dryer (dehydrator for vegetables, model MSG-01, MPM Product, Milanówek, Poland) and dried at 65 °C to constant mass. Dried fungal materials were ground using porcelain pestles and mortars that were cleaned by hand washing using laboratory brush, deionized water and detergent and further rinsed with distilled water and dried in an electrically heated laboratory dryer at 105 °C, and next were transferred into screw-capped plastic tubes (VWR®, Ultra High Performance, 15 mL), then sealed in polyethylene bags and kept in dry and clean condition in a storage room until analysis. These fungal materials were considered as the reference samples for household-treated mushrooms (Falandysz and Drewnowska, 2017).

### Frozen mushrooms

Two subsamples of the fruiting bodies of *C*. *cibarius* were divided into portions and kept frozen at −20 °C for 1 month. Frozen mushrooms were further blanched or blanched and pickled.

### Blanching

Fresh or frozen mushrooms were blanched respectively for 5 to 15 min using gently boiling distilled water (150 mL) in glass bakers or with mineral water (150 mL) in a stainless steel pot. The mushroom to water volume in the experiments was as 1:5. After blanching, mushrooms were drained and the subsamples were taken and freeze-dried, ground and kept in screw-capped plastic tubes in sealed polyethylene bags for trace elements determination.

### Pickling

The blanched *C*. *cibarius* samples were pickled using a vinegar-based marinade. The spirit vinegar of 10% acidity (in glass bottle with an aluminium screw cap and polyethylene gasket, 0.5 L) used for preparation of the marinade was bought in a grocery shop. The marinade was made by dilution of a vinegar respectively with deionized and potable water in proportion 1:4. Mushrooms were pickled in glass bakers (150 mL). Bakers filled with mushrooms and marinade were tightly sealed using a plastic foil from the top and kept in room temperature for 1 month. Next, mushrooms were drained, lyophilized and ground—using porcelain pestle and mortar. Grounded mushrooms were packed into screw-capped plastic tubes (VWR®, Ultra High Performance, 15 mL). Tubes with a fungal material were further packed into a foil bag, which was sealed, and kept in dry and clean condition in a storage room until further analysis.

### Dry biomass determination

Dry biomass content was determined for each sample of mushrooms—fresh and processed frozen, blanched and pickled (PN-90A-7510/03, 2003).

### Spectroscopic analyses

Cadmium in fungal materials from all treatments and processes was determined using validated methods by inductively coupled plasma mass spectrometry with dynamic reaction cell (ICP-DRC-MS, ELAN DRC II ICP-MS) (Falandysz et al. 2017a). The fungal materials were wet-digested with a solution of concentrated nitric acid in pressurized vessels made of poly-tetrafluoroethylene (PTFE) with aid of microwaves. In the same way, several certified reference materials of known content of Cd and the procedural blanks were examined (Table 1) (Falandysz et al. 2017a). The method limit of quantification (LOQ) of Cd in aqueous solution by the ICP-MS measurement was 1 μg/l.

**Table 1:** Results of the measurements of accuracy of the analytical data using certificate reference materials “fungal-powdered fruiting bodies of *Leccinum scabrum*” IC-CS-M-4 (*n* = 5); Oriental basma tobacco leaves, INCT-OBTL-5 (*n* = 5); and mixture of Polish herbs, INCT-MPH-2 (*n* = 5) by ICP-DRC-MS

Analyte	Reference material	Measured value (mg kg^−1^)	Certified value (mg kg^−1^)	Recovery (%)
Cd	IC-CS-M-4	1.43 ± 0.13	1.33 ± 0.09	107
Cd	INCT-TL-1	0.030 ± 0.004	0.030 ± 0.006	101
Cd	INCT-MPH-2	0.199 ± 0.015	0.194 ± 0.008	97

## Results and discussion

### Cadmium in *C*. *cibarius*

The cadmium concentrations in *C*. *cibarius* in this study were in range 0.17 ± 0.01 to 0.27 ± 0.01-mg kg^−1^ dry biomass (db). Cadmium concentrations in *C*. *cibarius* from the sites across Europe usually not exceeded a value of 1 mg kg^−1^ db, while when foraged from the montane sites somewhere or from the sites impacted by the metal smelting activities showed concentrations above 1 mg kg^−1^ db (Drewnowska and Falandysz 2015; Falandysz and Drewnowska 2015; Falandysz et al. 2012).

### Loss of biomass in process of blanching and pickling

Conventional drying of fresh mushrooms at 65 °C to constant biomass removes most of water and some well volatile organic components, while freeze-drying basically removes moisture. In this study, fresh and sliced fruiting bodies of *C*. *cibarius* when blanched lost on the average 36% of biomass, while when they were further pickled they lost in total 40% of the original biomass. Frozen fruiting bodies of the *C*. *cibarius* when blanched lost 62% of biomass, while when further pickled they lost 74% of biomass (largely water and water soluble compounds).

### Impact of blanching and pickling on content of cadmium in *C*. *cibarius*

Blanching of sliced fresh fruiting bodies of *C*. *cibarius* caused decrease of the Cd content by 11 ± 7 to 36 ± 7% and of sliced frozen fruiting bodies by 40 ± 6%. In the next treatment step, the pickling of initially blanched fresh fruiting bodies caused further loss of cadmium in the range of 42–71%, median values (*p* < 0.05, Mann-Whitney *U* test; Table 2). Total rate of Cd leaching from the blanched and further pickled *C*. *cibarius* was 72–91%, median values.

**Table 2:** Cadmium content and its leaching rates from the household-treated *C*. *cibarius*

Initial status before drying, blanching and further pickling	Cd content (mg kg^−1^ db)		Treatment time and type of water	Decrease of Cd (%)	Cd content (mg kg^−1^ db)	Estimated rate of Cd decrease in relation to blanched mushrooms (%)	Total decrease of Cd (%)
	Dried mushrooms	Blanched mushrooms^b^			Pickled mushrooms^c^		
Fresh; 2 (112)^a^	0.22 ± 0.03^d^ 0.22 0.17–0.25	0.14 ± 0.01 0.14 0.13–0.15	5 min; deionized	36 ± 7 39 25–43	0.046 ± 0.013 0.050 0.025–0.061	43 ± 8 40 35–54	78 ± 6 76 73–90
Fresh; 2 (311)	0.18 ± 0.04 0.19 0.12–0.23	0.13 ± 0.03 0.13 0.095–0.16	5 min; mineral	28 ± 9 27 18–41	0.035 ± 0.009 0.031 0.027–0.050	53 ± 8 57 42–58	81 ± 4 82 75–86
Fresh; 3 (267)	0.21 ± 0.04 0.19 0.17–0.27	0.17 ± 0.03 0.17 0.13–0.23	15 min; mineral	17 ± 9 16 7.1–32	0.024 ± 0.012 0.026 0.0059–0.044	71 ± 11 67 60–89	88 ± 7 89 73–97
Fresh; 2 (331)	0.27 ± 0.01 0.26 0.26–0.28	0.19 ± 0.07 0.22 0.095–0.25	15 min; deionized	11 ± 7 12 3.7–20	0.074 ± 0.032 0.076 0.039–0.11	61 ± 11 60 47–77	72 ± 11 72 60–85
Fresh; 3 (242)	0.34 ± 0.16 0.27 0.23–0.53	0.27 ± 0.13 0.23 0.17–0.41	15 min; deionized	20 ± 6 22 14–25	0.030 ± 0.004 0.029 0.027–0.035	70 ± 6 71 63–75	90 ± 3 89 88–93
Fresh; 3 (142)	0.29 ± 0.03 0.29 0.27–0.32	0.19 ± 0.01 0.19 0.18–0.20	15 min; mineral	35 ± 1 35 34–36	0.027 ± 0.013 0.024 0.016–0.041	56 ± 5 56 51–60	91 ± 4 91 87–94
Frozen; 6 (298)	0.53 ± 0.22 0.63 0.22–0.75	0.32 ± 0.13 0.37 0.12–0.46	15 min; deionized	40 ± 6 41 31–53	0.11 ± 0.03 0.11 0.047–0.15	38 ± 7 40 29–48	77 ± 7 80 64–85

^a^Number of composite samples and total number of fruiting bodies/caps (in parentheses)

^b^Kept for 5 or 15 min in gently boiling water

^c^They were blanched before pickling

^d^Mean ± SD, median and range

Duration of the blanching step, type of water used and status of the mushrooms used (fresh or frozen) were without impact on the total rate of Cd leaching, while blanching could be more effective for frozen (decrease at 41%, median value) than fresh (decrease in the range of 12–39%, median values) fruiting bodies. Also, in a study by Svoboda et al. (2002), it was noted that because of boiling under reflux of sliced fruiting bodies of *Imleria badia* (Fr.) Vizzini (former name *Xerocomus badius* (Fr.) E.-J. Gilbert) for 15 to 60 min, the rate of Cd leaching was higher from the frozen (58%) than from the fresh (36%) mushrooms, while time of boiling was without an impact.

In the case of the genus *Agaricus* mushrooms, pieces of a fresh *Agaricus blazei* Murrill from the farm, when boiled in a metal vessel for a period of 20 min leached 36% of Cd (Sun et al. 2012). A simple washing and deskinning of the fruiting bodies of *A. bisporus* in one study caused decrease of Cd content by 30–40% (Źródłowski 1995).

Cadmium forms complexes with protein-like structures in fruiting bodies of mushrooms. This metal was identified in fruiting bodies of *Agaricus macrosporus* (F.H. Møller and Jul. Schäff.) Pilát (current name *Agaricus urinascens* (Jul. Schäff. & F.H. Møller) Singer) in glycoprotein complex free of sulphur (Meisch and Schmitt, 1986). In mushroom *Agaricus augustus* Fr., Cd was in a large portion associated with water soluble ligands of a size from 55,000 to 100,000 Da (Lind et al. 1995).

The alkali elements such as potassium and caesium when compared with cadmium can be easier removed from mushrooms by simple cooking, this is because they are largely in protoplasm of the cells, form weaker bonds within the cell structures and their compounds are better water soluble. Blanching of mushrooms highly reduced content of radioactive caesium (^137^Cs and ^134^Cs) in fruitbodies (Skibniewska and Smoczyński 1999). In the case of *C*. *cibarius*, a simple washing of the halves of fruitbodies for about 45 s under flowing tap water before cooking increased the amount of liquid released from the mushrooms and enhanced removal of ^137^Cs and ^134^Cs by around 30% in cooked (with vegetable oil and using the cooking pan) mushrooms (Steinhauser and Steinhauser 2016). Nevertheless, if a tasty juice (made of a vegetable oil/butter and liquid released from the roasted fruitbodies of *C*. *cibarius*) is eaten (usually is not discarded) with bread, a possible exposure can remain at the same level. Blanching and draining of mushrooms before further cooking (with butter and using the cooking pan) can be an alternative.

Cadmium, like mercury, is a chalcophile (sulphur seeker) element but in relation to cadmium, pickling of blanched *C*. *cibarius* with a diluted vinegar marinade had only a minor, if any effect on removal of accumulated mercury and was without effect on blanched caps of *Amanita fulva* (Falandysz and Drewnowska, 2017). This probably can be explained due to difference in chemical forms observed between both elements in fruiting bodies. Mercury, which is often well accumulated by mushrooms, can form in their flesh hardly soluble inorganic compounds (HgSe, HgS) and metalorganic complexes (Vogel-Mikus et al. 2016) with relatively firm or very firm bonds.

As noted in this study, pickling of *C*. *cibarius* using a vinegar-based marinade was on the average more efficient in leaching of cadmium than blanching in 90–95 °C using potable and deionized water. A reason could be better solubility of the cadmium complexes in acidified marinade than in non-acidified water, and in a result higher removal rate of such complex than leached in a parallel, the other organic constituents.

Cadmium is a toxic element for which contents in food (also from wild and cultivated or agricultural sites) are regulated in the European Union (EC 2006, EC 2008, EC 2014). In the case of three species of cultivated fungi, i.e. champignon mushroom (*A*. *bisporus*), oyster mushroom (*Pleurotus ostreatus*) and shitake (*Lenintula eodes*) limit for Cd is 0.2-mg kg^−1^ fresh product. For “other mushrooms”, limit for Cd is 1.0-mg kg^−1^ fresh product. The consignments of *C*. *cibarius* in this study showed on low contamination with cadmium, which was in the total range of 0.022–0.075-mg kg^−1^ fresh biomass and medians in the range of 0.019–0.063-mg kg^−1^ fresh biomass (assuming moisture content at 90%). Hence, regardless of a more or less contamination with cadmium, content of this element in *C*. *cibarius* and probably also in other mushrooms can be highly decreased after processing such as blanching and pickling, and what can highly reduce its impact on the health of a consumer.
